# Constrictive Pericarditis: A Challenging Diagnosis in Paediatrics

**DOI:** 10.1155/2015/402740

**Published:** 2015-09-06

**Authors:** Mariana Faustino, Inês Carmo Mendes, Rui Anjos

**Affiliations:** ^1^Cardiology Department, Hospital Fernando Fonseca, IC 19, Amadora, 2720-276 Lisbon, Portugal; ^2^Pediatric Cardiology Department, Hospital de Santa Cruz, Avenida Professor Reinaldo dos Santos, Carnaxide, 2790-134 Lisbon, Portugal

## Abstract

Constrictive pericarditis is an uncommon disease in children, usually difficult to diagnose. We present the case of a 14-year-old boy with a previous history of tuberculosis and right heart failure, in whom constrictive pericarditis was diagnosed. The case highlights the need to integrate all information, including clinical data, noninvasive cardiac imaging, and even invasive hemodynamic evaluation when required, in order to establish the correct diagnosis and proceed to surgical treatment.

## 1. Introduction

Constrictive pericarditis is characterized by the appearance of signs and symptoms of right heart failure due to loss of pericardial compliance and restricting diastolic filling [1]. It results from a chronic inflammatory process involving the parietal and visceral pericardial layers [2]. Idiopathic pericarditis, cardiac surgery, and radiotherapy are the most common causes in developed countries. Incomplete drainage of purulent pericarditis is a rare entity that should also be considered, but tuberculosis is still the most prevalent cause in developing countries, particularly in paediatric population [3, 4].

Constrictive pericarditis is not common in children and therefore not readily recalled, and the clinical picture often seems unrelated to the heart, so it sometimes may represent a diagnosis challenge [5].

## 2. Case Presentation

We present the case of a 14-year-old boy, born and resident in Angola. At 7 years of age he had fatigue, cough, fever, and no weight progression.

At 11 years of age pulmonary tuberculosis was diagnosed, confirmed in sputum cultures. He received quadruple antituberculosis therapy for 6 months. As heart failure symptoms and signs arose, with systemic and pulmonary congestion, the child was started on diuretics and evacuated to a Pediatric Cardiology Department in Portugal. The clinical examination revealed weight and height below the 3rd percentile, blood pressure and heart rate within normal limits, mild tachypnea, pulmonary auscultation with decreased sounds at the right inferior third and left basal crackles, and nonpulsatile hepatomegaly and ascites; jugular venous pulse was congestive until mandible angle; however the M-shaped pattern (prominent x and y descendants) was not evident. No kussmaul sign was present. No heart murmurs, pericardial rub, or pericardial knock was documented.

**Figure 1:**
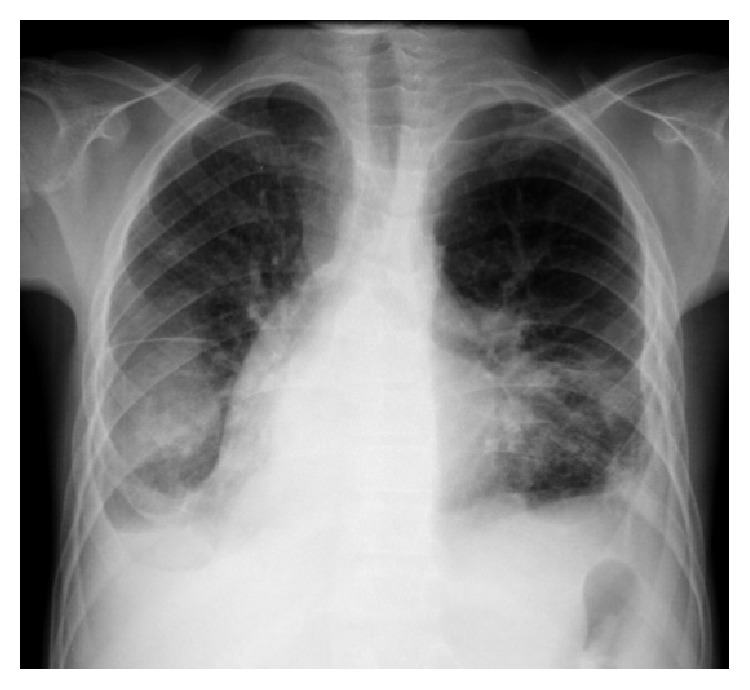
Posteroanterior chest radiography: mild cardiac dilation and bilateral pulmonary effusion, predominantly in the right hemithorax.

**Figure 2:**
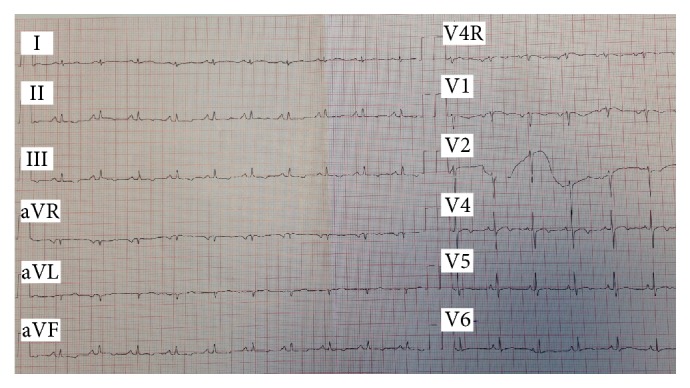
Twelve-lead electrocardiogram: heart rate of 90 beats/min; low voltage QRS in frontal leads; nonspecific alterations of ventricular repolarization.

A chest radiography showed mild cardiac dilation and bilateral pulmonary effusion, predominantly in the right hemithorax (Figure 1). Electrocardiogram showed low voltage QRS in frontal leads compatible with effusion and/or constriction process (Figure 2). Laboratory tests revealed mild hypoalbuminemia (2.9 mg/dL), with normal liver enzymes; serum inflammatory markers were negative. Viral and autoimmune investigation was also negative.

**Figure 3:**
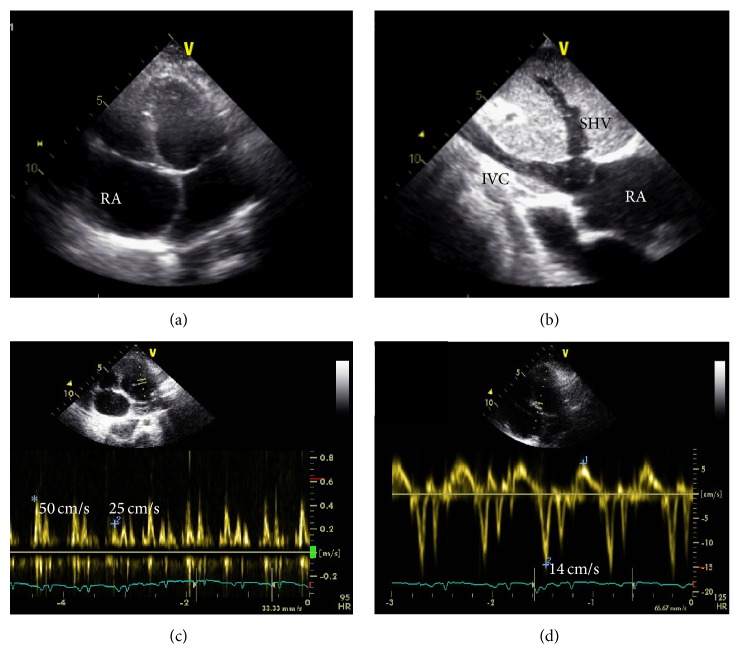
(a) Apical 4-chamber view with dilated right atria (RA), left deviated atrial septum; (b) subcostal view of dilated inferior vena cava (IVC) and suprahepatic veins (SHV); (c) significant (50%) transmitral flow variation with breathing, increasing during expiration (*∗*) and decreasing during inspiration (+); (d) high protodiastolic mitral annulus velocity (septal 14 cm/s, higher than lateral).

Echocardiography showed significant enlargement of the right atria, inferior vena cava, and suprahepatic veins (Figures 3(a) and 3(b), clip 1 in Supplementary Material available online at http://dx.doi.org/10.1155/2015/402740); right ventricular systolic pressure and function were normal; Left ventricle function and volumes were normal. There was no pericardial effusion, but signs of ventricular interaction were found, septal bounce and significant respiratory variation in transvalvular flow (transmitral flow increasing by 50% during expiration) (Figure 3(c)). Unfortunately, hepatic vein respiratory variation was not possible to adequately assess. Dilatation of the right chambers and the flow pattern through the atrio-ventricular valves, in the absence of pulmonary hypertension, nor systolic left ventricle dysfunction, suggested pericardial constriction. Protodiastolic mitral annulus velocity was preserved (*E*′ = 14 cm/s), and the ratio *E*/*E*′ was low (*annulus paradoxus*). Septal *E*′ velocity was higher than medial *E*′ velocity (*annulus reversus*). This findings were also suggestive of pericardial disease, and not restrictive cardiomyopathy (Figure 3(d)).

In the presence of this clinical history and physical examination, authors suspected constrictive pericarditis. Echocardiography parameters were also suggestive of constrictive physiology but however considered not enough to establish the diagnosis. For confirmation, magnetic resonance was performed. As the patient was uncooperative only anatomic imaging was performed, and constrictive physiology was not demonstrated.

**Figure 4:**
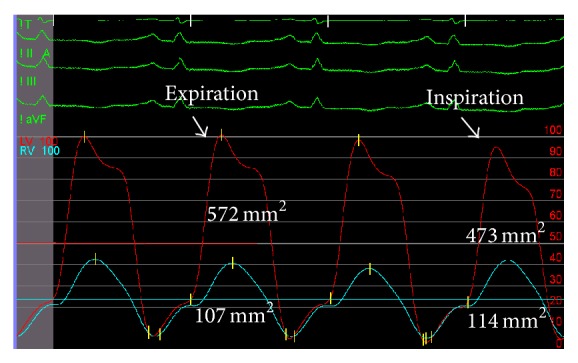
Elevated and equalized telediastolic pressure in ventricles (20 mmHg, arrow head); systolic area index calculated as the ratio between right ventricle/left ventricle systolic area in inspiration and right ventricle/left ventricle systolic area in expiration: (114/473)/(107/572) = 1.29.

In spite of a high clinical and echocardiographic suspicion, in order to confirm the diagnosis, before proposing a surgery with no negligible risks, cardiac catheterization was performed (Figure 4). Several parameters were suggestive of constriction: elevated and equalized telediastolic pressures in both ventricles; telediastolic pressure in right ventricle (20 mmHg) higher than one-third of its systolic pressure (42 mmHg); increased mean right atrial pressure (17 mmHg); systolic area index of 1.29, suggesting ventricular interdependence. The ventricular filling pattern was suggestive of the classic square root sign (dip and plateau).

**Figure 5:**
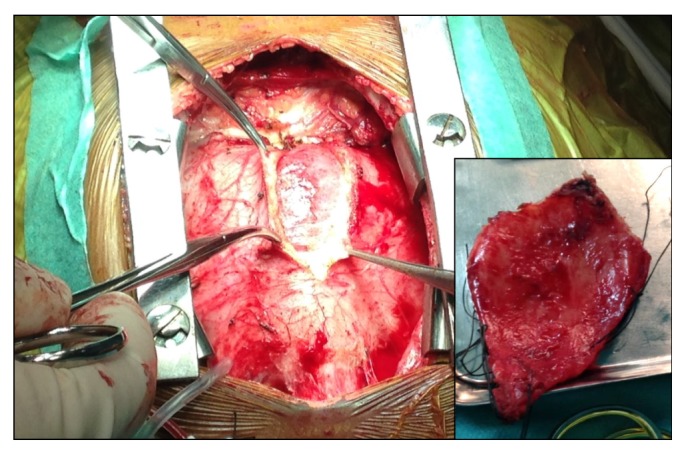
Thickened and adherent pericardium during and immediately after pericardiectomy.

Patient was referred for pericardiectomy. Pericardium was thickened and adherent, not calcified, and it was excised from phrenic to phrenic nerve (Figure 5). There was an immediate decrease in right atria mean pressure (25 to 12 mmHg). Pathologic study of the surgical specimen revealed extensive fibrosis without active inflammation, compatible with chronic fibrous constrictive pericarditis. We assume that Tuberculosis was the cause of the constrictive pericarditis. The postoperative period was uneventful, with fast and complete regression of all signs of heart failure.

## 3. Discussion

The long lasting symptoms of this patient were very likely related to tuberculosis, which progressed with pericardial involvement and constriction, not reversible with antituberculosis therapy.

Although constrictive pericarditis is rare in children, it should be considered in the differential diagnosis of right heart failure. It is possible that this low incidence in children is due, in part, to subdiagnosis and also to the insidious character of this disease and its complications [5].

A high index of suspicion is necessary to establish the diagnosis of constrictive pericarditis [5]. It is necessary to integrate clinical evaluation with data from the several available methods and to have a critical approach position when eventual discrepancy arises.

Transthoracic echocardiography is usually the first diagnostic investigation performed for suspected constrictive pericarditis and can be very useful in the presence of the typical findings: septal bounce, dynamic respiratory variation in flow (transtricuspid, transmitral, and hepatic vein), and enhanced ventricular interaction [3]. In this way echocardiography has an important role in the differential diagnosis of constrictive pericarditis with other causes of heart failure, namely, restrictive cardiomyopathy, the most challenging entity to differentiate from constrictive pericarditis [1, 6]. However echocardiography might not be confirmatory in all cases, since some of these typical findings may not be present [5].

Cardiac magnetic resonance, despite being the imaging method of choice to evaluate the pericardium, was not useful and potentially confounding in this patient. Contrast enhanced CT angiography, being also a noninvasive investigation, is more tolerable and easier to perform in children than cardiac magnetic resonance and could have been a better option for this patient. It is useful to identify pericardial thickness and effusion, to detect enlargement of the mediastinal lymph nodes, and to evaluate lung parenchyma, which may be important in patients suspected to have tuberculosis [3].

Cardiovascular catheterization, although an invasive method and not always necessary, is the gold standard for the diagnosis of constriction and was crucial to confirm the diagnosis. A recent described criteria is the systolic area index, ratio of right ventricle to left ventricle systolic area during inspiration and expiration, that predicts ventricular interdependence with 97% sensitivity and 100% positive predictive power, when higher than 1.1 [7].

The case highlights the need to integrate all information, including clinical data, electrocardiography, chest radiography, cardiac imaging data, and even invasive hemodynamic evaluation. Cardiac catheterization is usually reserved for patients in whom there is significant diagnostic doubt after noninvasive evaluation, since the confirmation of the diagnosis is decisive for the therapeutic approach, in order to ensure that the expected benefit outweighs the potential risk.

A correct and early diagnosis of constrictive pericarditis is extremely important, since successful pericardiectomy can be curative [3]. In advanced disease, surgery results are suboptimal and mortality is higher, which is also related to poor preoperative general condition and advanced preoperative NYHA class [3].

## Supplementary Material

Clip 1 (Optional Supplementary Materials): Echocardiography showing significant enlargement of the right atria, inferior vena cava and suprahepatic veins and interventricular septal bounce, suggesting ventricular interaction.
